# Integrating Multibeam Backscatter Angular Response, Mosaic and Bathymetry Data for Benthic Habitat Mapping

**DOI:** 10.1371/journal.pone.0097339

**Published:** 2014-05-13

**Authors:** Rozaimi Che Hasan, Daniel Ierodiaconou, Laurie Laurenson, Alexandre Schimel

**Affiliations:** 1 Deakin University, Centre for Integrative Ecology, School of Life and Environmental Sciences, Faculty of Science and Built Environment, Warrnambool, Victoria, Australia; 2 UTM Razak School of Engineering and Advanced Technology, Universiti Teknologi Malaysia, Kuala Lumpur, Malaysia; University of Auckland, New Zealand

## Abstract

Multibeam echosounders (MBES) are increasingly becoming the tool of choice for marine habitat mapping applications. In turn, the rapid expansion of habitat mapping studies has resulted in a need for automated classification techniques to efficiently map benthic habitats, assess confidence in model outputs, and evaluate the importance of variables driving the patterns observed. The benthic habitat characterisation process often involves the analysis of MBES bathymetry, backscatter mosaic or angular response with observation data providing ground truth. However, studies that make use of the full range of MBES outputs within a single classification process are limited. We present an approach that integrates backscatter angular response with MBES bathymetry, backscatter mosaic and their derivatives in a classification process using a Random Forests (RF) machine-learning algorithm to predict the distribution of benthic biological habitats. This approach includes a method of deriving statistical features from backscatter angular response curves created from MBES data collated within homogeneous regions of a backscatter mosaic. Using the RF algorithm we assess the relative importance of each variable in order to optimise the classification process and simplify models applied. The results showed that the inclusion of the angular response features in the classification process improved the accuracy of the final habitat maps from 88.5% to 93.6%. The RF algorithm identified bathymetry and the angular response mean as the two most important predictors. However, the highest classification rates were only obtained after incorporating additional features derived from bathymetry and the backscatter mosaic. The angular response features were found to be more important to the classification process compared to the backscatter mosaic features. This analysis indicates that integrating angular response information with bathymetry and the backscatter mosaic, along with their derivatives, constitutes an important improvement for studying the distribution of benthic habitats, which is necessary for effective marine spatial planning and resource management.

## Introduction

Marine biodiversity worldwide is under pressure from a wide variety of anthropogenic activities [1], [2]. The mapping of marine habitats is viewed as the first step in the process of studying, managing, protecting and ultimately conserving marine biodiversity [3]. Multibeam echo sounders (MBES) are now extensively used for this purpose, chiefly because they present technological capabilities (swath coverage, acquisition of high-resolution bathymetry, wide depth range) that all other existing systems, such as single-beam echo sounders, side-scan sonars or Light Detection And Ranging (LiDAR), fail to combine [4]. Various methods of classifying MBES data into habitat maps have been developed over the past two decades. These methods vary widely in terms of the classification algorithms that are implemented, but also in the data features used for classification. There are three types of MBES datasets commonly used as features and/or sources of derivative features for the classification process: backscatter mosaic, backscatter angular response and bathymetry.

A MBES backscatter mosaic is a georeferenced grey-level image representing the acoustic intensity scattered by the seabed, with different seabed types usually showing different intensity levels [5]. Since the acoustic intensity scattered by the seabed is varying with the angle of incidence of the acoustic signal at the seafloor at the time of data acquisition, a statistical normalization of this angular variation is required prior to forming the backscatter mosaic, so that the intensity variations in the image are due to geographical changes in seafloor-type only [6]. This normalization process implies that the quantitative aspect of the intensity level is lost, so that any analysis of the resulting backscatter mosaic requires some form of qualitative interpretation or ground-truthing [7]. The backscatter mosaic grey-level has been extensively used as a feature in many classification techniques [8]–[11] or as a source of derivative features describing, among other image characteristics, the grey-level statistics [12], [13] or the texture [14].

The MBES backscatter angular response is the acoustic intensity scattered by the seabed as a function of the angle of incidence of the acoustic signal at the seafloor. Often represented as the mean angular curve, the backscatter angular response is characteristic of the type of seafloor that reflected the acoustic signal [7]. Since the angular response is not normalized like the backscatter mosaic, it potentially allows the extraction of quantitative seafloor characteristics [7]. Forming a useful mean angular response curve requires the collection of several data samples from the widest angular range possible. In practice, this is obtained by combining several consecutive pings over a full or half swath, which leads to a spatial resolution that is considerably coarser compared to that achieved in the backscatter mosaic format. Furthermore, the large area of seabed thus covered might not present a homogenous seabed type, and thus lead to errors in the angular response analysis. As a consequence, approaches based on exploiting features describing the backscatter angular response curves have remained relatively scarce to date in comparison to those exploiting the backscatter mosaic format [4]. However there has been a renewed interest in this type of analysis recently, with a number of studies testing a number of different features for their predictive power [15]–[19].

Bathymetry is the data type MBES were originally designed to record. Bathymetry is a major driver of species distributions in coastal waters as depth influences the amount of light reaching the seafloor and exposure to wave action and tide induced currents. In addition, full-coverage bathymetry allows the extraction of seascape metrics that may be used to estimate variations in environmental complexity, which might influence the area available for settlement, food and protection from predation [20]. The predictive power of MBES bathymetry data and their derivative metrics in revealing habitat spatial distribution patterns and the relationship between seabed type and benthic habitats has often been demonstrated [21]–[24].

The past decade has seen an increase in classification techniques developed to exploit features commonly derived from two of these three MBES data sources. Methodologies that integrate both bathymetry derivatives and backscatter mosaic features have become commonplace and have shown improvements in class separation and overall classification success [10], [25]–[29]. In parallel, Fonseca et al. [30] suggested that backscatter mosaic and angular response should be combined to use both the fine spatial resolution of the former and the predictive power of the latter. Although promising, this suggestion has rarely been implemented [31], [32]. Finally, there has been attempts at integrating features extracted from bathymetry and angular response curves although they have remained scarce to date [33]. However, to our knowledge, no benthic habitat classification methodology has been designed to integrate features derived from all three MBES data sources. Given the improvements in classification accuracy obtained by the more recent methodologies combining two datasets over the more traditional methodologies that only exploit one, it can be expected that the integration of features extracted from the three data sources could further improve the class-differentiation process.

Irrespective of whether existing habitat-mapping classification techniques focus on backscatter mosaic, backscatter angular response or bathymetry data (or their integration), those that exploit a set of several data features often fail to assess which of these features contribute the most to classification success. With an increasing number of classification approaches being available that use an increasing number of features derived from MBES data, future classification efforts should be accompanied with the identification of which features are the most relevant to classification success. This issue is becoming particularly pressing with the increasing volume of data and the growing demand for mapping products [4]. Random Forests (RF) may address this specific requirement, as they provide a measure of relative importance for each feature as a complement to their classification output. Typically, an RF algorithm works by training several decision trees, and combining their results through a voting process with the number of trees set by the user, and each tree voting for a particular class [34]. Contrary to standard decision tree algorithms that split nodes based on the best split amongst all variables, RF algorithms split nodes by using the best among a subset of predictors that are randomly chosen at each node [35]. The capability of an algorithm to estimate the relative importance of each feature stems from this random subset selection process. RF algorithms have repeatedly proven successful in predicting fish assemblages [36], in mapping near shore epi-macrobenthic species richness from airborne LiDAR data [37] and in mapping benthic habitats from Autonomous Underwater Vehicle (AUV) images [38].

Accordingly, the objectives of the present study are to integrate angular response features with standard products derived from both bathymetry and backscatter mosaic and assess whether this integration lead to increased classification accuracy, using the capability of the RF algorithm to estimate the relative importance of each feature.

## Methods

### Ethics statement

**Figure 1:**
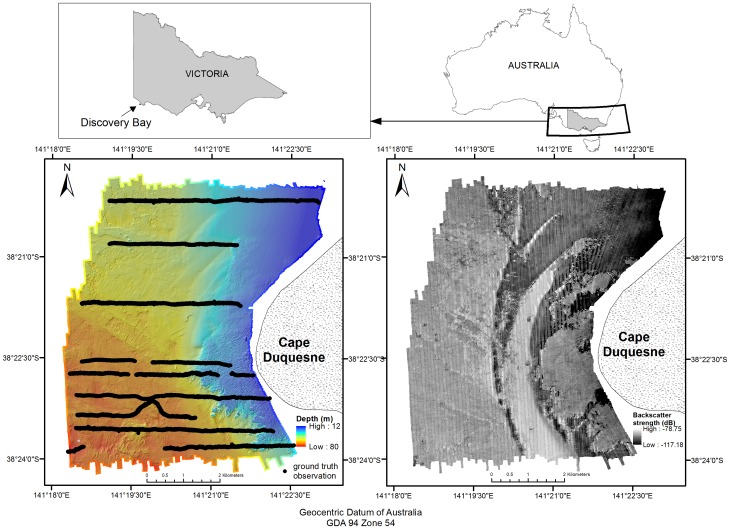
Study site location (top panels), bathymetry grid with superimposed ground truth observation transects (bottom left panel) and backscatter mosaic (bottom right panel).

The remotely sensed techniques used in this study (both sonar and video observations) did not require a permit for their use in survey, thus no specific permission were sought for data collection in the study area location (GPS coordinates can be found on Figure 1). The field studies did not involve endangered or protected species.

### Study area

The study site encompassed a 42 km^2^ area, with sea depths ranging from 11 to 80 m, located off Cape Duquesne in Discovery Bay, in the state of Victoria, south-eastern Australia (Figure 1). Shallow reefs in this area support diverse assemblages of red algae and kelps (dominated by *Ecklonia radiata, Phyllospora comosa* and *Durvillaea potatorum*), while deeper reefs are dominated by invertebrate communities with sponges, ascidians, bryozoans and gorgonian corals [39].

### Acoustic data acquisition

The acoustic data were acquired on the 6th and 7th of November 2005 using a hull-mounted Reson SeaBat 8101 MBES, integrated with a Fugro “Starfix.HP” Differential GPS system for positioning (±0.30 m accuracy) and an Applanix POS MV (Position and Orientation Systems for Marine Vessels) for heave, pitch, roll and yaw corrections (±0.02° accuracy). Real-time navigation, data-logging, quality control and display were provided by the Fugro Starfix suite 8.1 software. Daily sound velocity profiles were collected to correct the acoustic data for water-column sound speed variations.

### Bathymetry map and derivatives

Depth soundings were cleaned using the Fugro Starfix suite, reduced to the lowest astronomical tide datum using tidal observations, and gridded to produce a bathymetric grid at 2.5 m resolution (Figure 1). Six derivative layers were produced from the high-resolution bathymetry grid using various GIS software (Table 1); aspect, rugosity, maximum curvature, bathymetric position index (BPI), slope and complexity [20], [24], [40]. These six layers were selected based on their successful application in thematic benthic habitat map construction of previous studies [25]–[27].

**Table 1:** Detailed explanations of all layers derived from bathymetry and the backscatter mosaic.

Derivatives (original layer)	Details	Analysis window (pixel size: 2.5 m)	Software
Aspect (Bathymetry)	Describes the azimuthal direction of the steepest slope through the points in the analysis window [20]	3×3	Spatial Analyst (ArcGIS 9.3)
Rugosity (Bathymetry)	A measure of structural complexity represented by the ratio of surface area to planar area [24]	3×3	Benthic Terrain Modeller Tool for ArcGIS
Maximum curvature (Bathymetry)	Describes the steepest curve of either plan or profile convexity through a defined cell neighbourhood [40]	3×3	ENVI 4.7
Bathymetric Position Index (Bathymetry)	Compares the elevation of each cell in a digital elevation model to the mean elevation of a specified neighbourhood around that cell [68]	Inner radius = 10, Outer radius = 10, Scale factor = 125	Benthic Terrain Modeller Tool for ArcGIS
Slope (Bathymetry)	Describes the maximum change in elevation between each cell and cells in the analysis neighbourhood [20]	3×3	Spatial Analyst (ArcGIS 9.3)
Complexity (Bathymetry)	Describes the rate of change in the bathymetry slope [20]	3×3	ENVI 4.7
Red, Green and Blue bands of Hue, Saturation and Intensity (Backscatter mosaic)	High and low frequency information of an image after application of high and low pass filters, producing three band images of Red, Green and Blue [43].	3×3 (high pass filter) and 11×11 (low pass filter)	ENVI 4.7
Homogeneity, Entropy and Correlation texture features (Backscatter mosaic)	Texture features calculated from Grey Level Co-occurrence Matrices (GLCM) [44]	7×7	ENVI 4.7

### Backscatter mosaic and derivatives

The backscatter mosaic was obtained using the CMST MB Process software v10.04.04.2, developed by Curtin University's Centre for Marine Science and Technology [41], [42]. First, vessel attitude data (i.e. roll, pitch, yaw, heave and heading) and sounding slant range were used to estimate the actual depth and location of measurements within each beam in every ping. Then, raw signal amplitude data were reduced to seafloor backscatter intensity using radiometric corrections, including TVG (Time-Varying Gain) removal, the estimation of spreading and absorption losses and the compensation for the beam footprint size. The angular dependence was then statistically compensated using a ‘sliding window’ of 25 consecutive pings and a reference angle of 30°. Finally, a backscatter mosaic was produced at 2.5 m resolution and exported for further analysis (Figure 1).

Six derivatives were produced from the backscatter mosaic using the ENVI 4.7 software (Table 1): specifically, Red, Green and Blue layers of Hue, Saturation and Intensity (HSI) [43], and the Haralick texture features Homogeneity, Entropy and Correlation, calculated from Grey Level Co-occurrence Matrices (GLCM) [44]. Like the bathymetry derivatives, the three HSI layers were selected based on their ability to produce accurate benthic habitat maps in previous studies [25]–[27]. Homogeneity, Entropy and Correlation were selected, among a wide range of other texture features available [44], based on their reported importance in previous texture-based habitat mapping efforts [14], [28], [45], [46] and on their belonging to three different groups, so as to minimise risks of correlation [47]. The three texture features were obtained by calculating the GLCMs in the 0°, 45°, 90° and 135° directions over the 8-bit backscatter mosaic (with no greyscale normalisation applied), extracting the features from each GLCM direction, and averaging the results.

### Angular response derivatives

**Figure 2:**
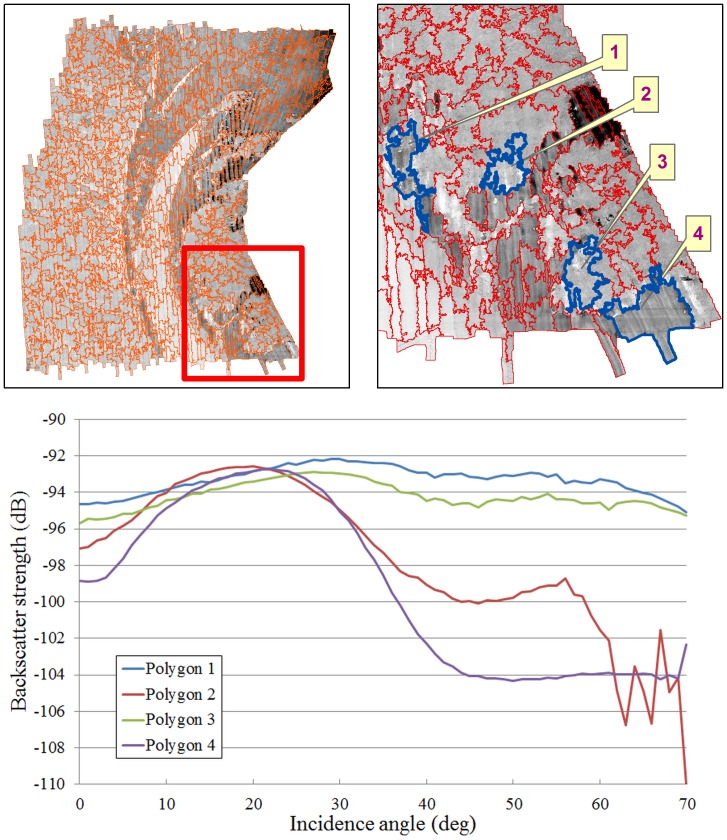
(a) Backscatter mosaic overlaid with the results of the region-growing segmentation. (b) Detailed view of the mosaic segmentation as indicated by the red box in (a). (c) Examples of four different angular response curves computed from polygons 1–4 as indicated by the blue sections in (b).

The angular response curves were obtained by combining the seafloor backscatter intensity samples produced by the MB Process software (prior to the statistical angular compensation that leads to the mosaic) and a segmentation of the mosaic. First, the mosaic was segmented into separate contiguous regions using a region-growing algorithm in Spring v5.1 software. By initially using each mosaic pixel as a distinct region (“seeds”), the algorithm recursively aggregated the neighbouring regions presenting the maximum grey-level similarity, as long as this maximum similarity fell under a user-defined similarity threshold that became increasingly less stringent as the algorithm progressed [48]. At the end of this recursive process, a second user-defined area threshold that specified a minimum region-size, allowed the smallest regions to be aggregated with larger adjacent regions. For this study, a similarity threshold of 1 and an area threshold of 2500 were used as parameters to produce the segmentation. The segmentation was then imported into ArcMap, in which all segments were vectorised as polygons (Figure 2a). Finally, using proprietary Matlab code (available in Supplementary S1), the seafloor backscatter intensity samples and their associated angle of incidence were compiled for all MBES data files over each polygon, and the mean intensity value (in dB space) for each angle was computed. This process resulted in a single backscatter angular response curve for each segment (Figure 2b and c).

**Figure 3:**
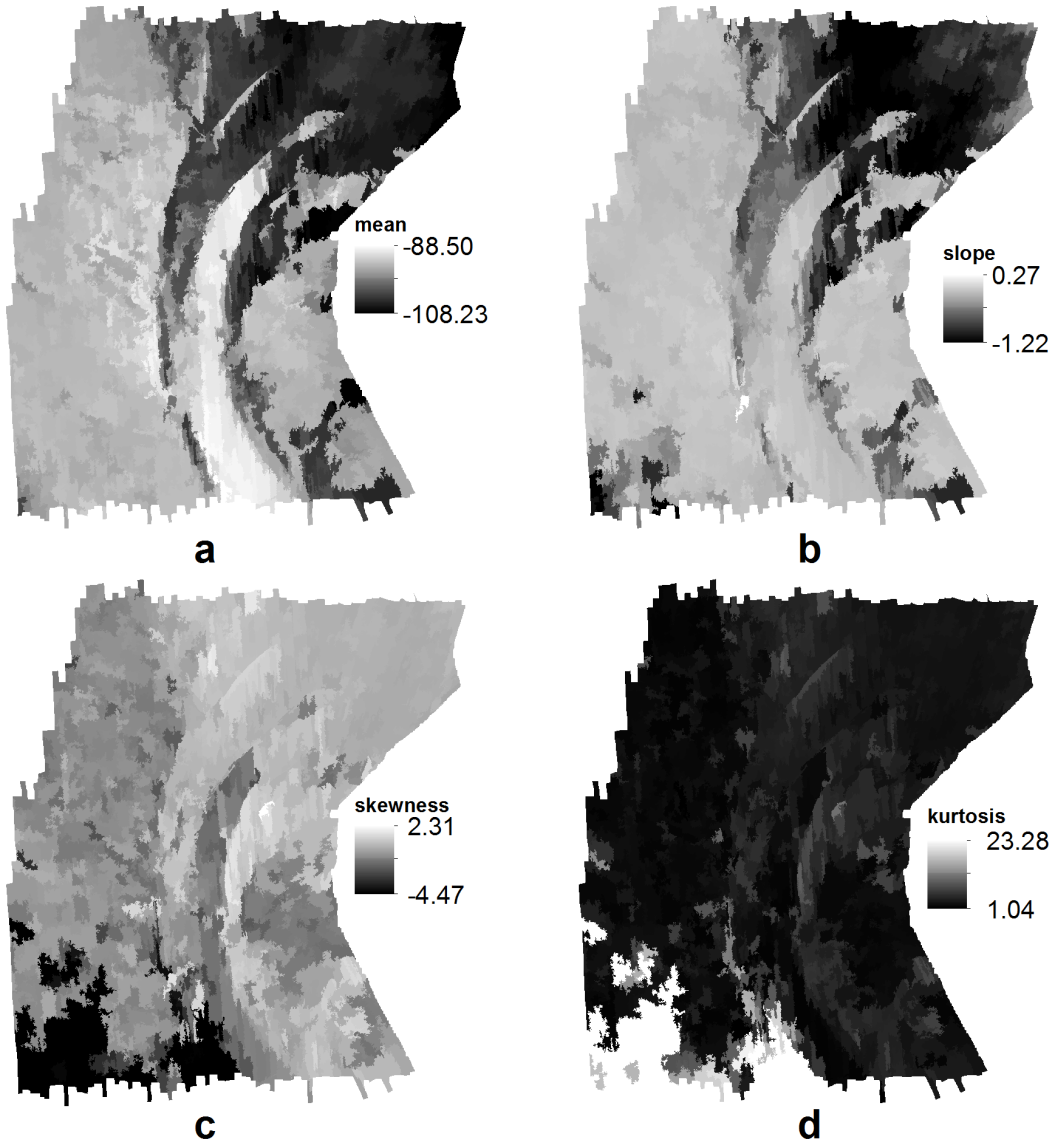
Maps of the angular response features, derived from the backscatter angular response curves between 30° and 50°; (a) mean, (b) least square slope, (c) skewness and (d) kurtosis.

Four derivatives were produced from the backscatter angular response curves using Matlab: mean, least square slope, skewness and kurtosis of the backscatter intensity within 30 to 50° incidence angles. The derivative values were then attributed to their respective polygons, and rasterised at a resolution of 2.5 m using ArcMap (Figure 3).

### Data layer correlation analysis

The 18 data layers that were obtained from the acoustic data (bathymetry + six bathymetry derivatives + mosaic grey-level + six mosaic derivatives + four angular response derivatives) were tested for correlation in ENVI 4.7, through the computation of Pearson's linear correlation coefficient (*R^2^*).

### Towed video observations

A VideoRay PRO 3 GTO Remotely Operated Vehicle (ROV) was used to provide ground truth information for model building and evaluation (Figure 1). Underwater acoustic positioning of the towed video system was achieved by using a Tracklink 1500MA Ultra Short Base Line (USBL) acoustic tracking system, with vessel errors (roll, pitch and yaw) being corrected for by using a KVH Industries motion sensor. An Omnistar wide-area Differential Global Positioning System (DGPS) was used to fix the vessel location, and apply corrections for acoustically positioned video (±2.5 m accuracy). The recorded video data were classified according to the Victorian Towed Video Classification scheme to identify benthic biota classes. The classification scheme followed the guidelines published by the Interim Marine and Coastal Regionalisation for Australia [49]. Seven habitat classes were identified from the video observations; Mixed Brown algae (MB), Invertebrates (INV), Mixed Red algae and Invertebrates (MRI), Mixed Brown algae and Invertebrates (MBI), Mixed Brown algae and Mixed Red algae (MBMR), Mixed Green algae and Invertebrates (MGI) and No Visible Biota (NVB). All available reference data were randomly sampled for model development (70%) and for the accuracy assessment (30%) (Table 2).

**Table 2:** Number of samples used for model development and accuracy assessment, for each biota class.

Biota class	Number of samples used for model development	Number of samples used for accuracy assessment
Mixed Brown algae (MB)	1107	475
Invertebrates (INV)	11830	5070
Mixed Red algae and Invertebrates (MRI)	1391	596
No Visible Biota (NVB)	11915	5107
Mixed Brown algae and Invertebrates (MBI)	593	254
Mixed Brown algae and Mixed Red algae (MBMR)	62	26
Mixed Green algae and Invertebrates (MGI)	749	321

### Random Forests

Supervised RF decision trees were implemented to train and subsequently model class predictions. A RF algorithm programmed in Matlab was used [50], combined with a proprietary Matlab routine (available in Supplementary S1) developed to read and process multilayer images in native ENVI format. For this study, the RF algorithm parameters *m* (number of predictors randomly chosen for each split) and *ntree* (number of trees generated) were set to the integral part of the square root of the total number of variables (default setting) and 200 (to minimise errors rates) respectively.

Initially, two RF models were generated to test for the relevance of the angular response derivatives (Table 3). The first model was limited to bathymetry and the backscatter mosaic and their derivatives (Model 1), while the second model also incorporated the angular response derivatives (Model 2). The contribution of each input layer to each of these two models was ranked by importance (scaled from 0 to 1). Additional RF models were then generated to test whether the success rate of these initial models could be achieved by using fewer input layers. First, an RF model was generated using only the input layers with an importance score of 1 (Model 3). Then, additional RF models were generated, in which the input layers of lesser importance were gradually added, based on two rules: (1) three or less variables should be added at a time, and (2) the differences in the importance score between the added variables should be less than 0.2 (Models 4 to 6). The accuracy of each model was assessed by forming an error matrix, and computing its overall accuracy and Kappa coefficient [51]. Z statistics were computed from pairwise combinations of the error matrices to compare the model outputs [52].

**Table 3:** Variable combinations and classification accuracy results for the different models.

Model number			#1	#2	#3	#4	#5	#6
Total number of variables in model			14	18	2	5	8	11
Model variables	Bathymetry		√	√	√	√	√	√
	Bathy derivatives	Complexity	√	√				√
		Aspect	√	√				
		BPI	√	√				
		Slope	√	√				
		Maximum Curvature	√	√				
		Rugosity	√	√				√
	Mosaic		√	√				
	Mosaic derivatives	Red HSI	√	√			√	√
		Green HSI	√	√			√	√
		Blue HSI	√	√			√	√
		GLCM Homogeneity	√	√				
		GLCM Entropy	√	√				
		GLCM Correlation	√	√				√
	AR derivatives	AR mean		√	√	√	√	√
		AR slope		√		√	√	√
		AR skewness		√		√	√	√
		AR kurtosis		√		√	√	√
Model performance	Overall accuracy (%)		88.5	93.6	90.2	89.7	92.2	93.5
	Kappa coefficient		0.81	0.90	0.84	0.83	0.88	0.90

The tick symbol (√) indicates the variable was used in a model.

## Results

### Backscatter angular response derivatives

The angular response mean (Figure 3a) was visually very similar to the backscatter mosaic (Figure 1), but presented two peculiarities. First, it showed more contrast and discrimination between the low and high backscatter regions of the study site. Second, it did not present the along-track artefacts that were still highly visible in the mosaic, despite statistical compensation. Similar observations were made for the angular response slope, although the low/high backscatter discrimination was less distinct (Figure 3b). The angular response skewness and kurtosis maps (Figure 3c and 3d) were very similar to each other, but showed little resemblance to the backscatter mosaic, the angular response mean and the angular response slope maps. In particular, the skewness and kurtosis maps appeared to highlight areas in the south-west of the study site that were not evident in the backscatter mosaic.

### Correlation of the layers

Strong auto-correlation (>0.5) was found among several layers derived from backscatter data, but not among bathymetry products (Table 4). The highest *R^2^* was found between the GLCM Homogeneity and Entropy layers (0.98). Confirming the visual analysis in the previous section, a high correlation was found between the angular response mean and angular response slope (0.75), between the angular response skewness and angular response kurtosis (0.72) and between the mosaic and the angular response mean (0.53). In comparison, the maximum *R^2^* measured between two layers derived from the bathymetry was relatively low (0.43; between complexity and bathymetry slope). Relatively low correlation was also observed between the original backscatter mosaic and bathymetry (0.33).

**Table 4:** Correlation of determination (*R^2^*) using the linear Pearson correlation measure between all variables used in this study.

Variables	Bathy	Comp	Asp	BPI	Slo	Maxc	Rug	Mos	HSIR	HSIG	HSIB	Hom	Ent	Cor	ARmean	ARslo	ARsk	ARkur
Bathy	1	-	-	-	-	-	-	-	-	-	-	-	-	-	-	-	-	-
Comp	0.00	1	-	-	-	-	-	-	-	-	-	-	-	-	-	-	-	-
Asp	0.06	0.02	1	-	-	-	-	-	-	-	-	-	-	-	-	-	-	-
BPI	0.00	0.03	0.00	1	-	-	-	-	-	-	-	-	-	-	-	-	-	-
Slop	0.00	0.43	0.00	0.03	1	-	-	-	-	-	-	-	-	-	-	-	-	-
Maxc	0.00	0.18	0.00	0.08	0.34	1	-	-	-	-	-	-	-	-	-	-	-	-
Rug	0.00	0.13	0.00	0.01	0.38	0.20	1	-	-	-	-	-	-	-	-	-	-	-
Mos	0.33	0.01	0.01	0.00	0.00	0.00	0.00	1	-	-	-	-	-	-	-	-	-	-
HSIR	0.24	0.00	0.01	0.00	0.01	0.00	0.00	0.18	1	-	-	-	-	-	-	-	-	-
HSIG	0.26	0.04	0.03	0.00	0.01	0.00	0.00	0.32	0.35	1	-	-	-	-	-	-	-	-
HSIB	0.06	0.05	0.02	0.01	0.02	0.01	0.00	0.10	0.08	0.08	1	-	-	-	-	-	-	-
Hom	0.10	0.01	0.01	0.00	0.00	0.00	0.00	0.33	0.07	0.00	0.28	1	-	-	-	-	-	-
Ent	0.11	0.01	0.01	0.00	0.00	0.00	0.00	0.36	0.04	0.00	0.23	**0.98**	1	-	-	-	-	-
Cor	0.05	0.01	0.00	0.00	0.01	0.00	0.00	0.09	0.12	0.05	0.07	0.00	0.00	1	-	-	-	-
ARmean	0.38	0.07	0.05	0.00	0.02	0.01	0.00	**0.53**	0.33	0.47	0.03	0.11	0.13	0.11	1	-	-	-
ARslo	0.26	0.11	0.05	0.01	0.04	0.01	0.00	0.24	0.22	0.36	0.02	0.02	0.02	0.04	**0.75**	1	-	-
ARsk	0.22	0.01	0.02	0.00	0.00	0.00	0.00	0.10	0.14	0.11	0.00	0.01	0.01	0.03	0.19	0.06	1	-
ARkur	0.10	0.00	0.00	0.00	0.00	0.00	0.00	0.01	0.03	0.01	0.00	0.00	0.00	0.00	0.01	0.00	**0.72**	1

Highlighted values indicate high correlation (*R^2^*>0.5). Bathy  =  Bathymetry, Comp  =  Complexity, Asp  =  Aspect, BPI  =  Bathymetric Position Index, Slo  =  Bathymetry slope, Maxc  =  Maximum curvature, Rug  =  Rugosity, Mos  =  Backscatter mosaic, HSIR  =  Red layer of Hue Saturation and Intensity (HSI), HSIG  =  Green layer of HSI, HSIB  =  Blue layer of HSI, Hom  =  GLCM Homogeneity, Ent  =  GLCM Entropy, Cor  =  GLCM Correlation, ARmean  =  Mean of angular response, ARslo  =  Slope of angular response, ARsk  =  Skewness of angular response and ARkur  =  Kurtosis of angular response.

### Variable importance and feature selection

**Figure 4:**
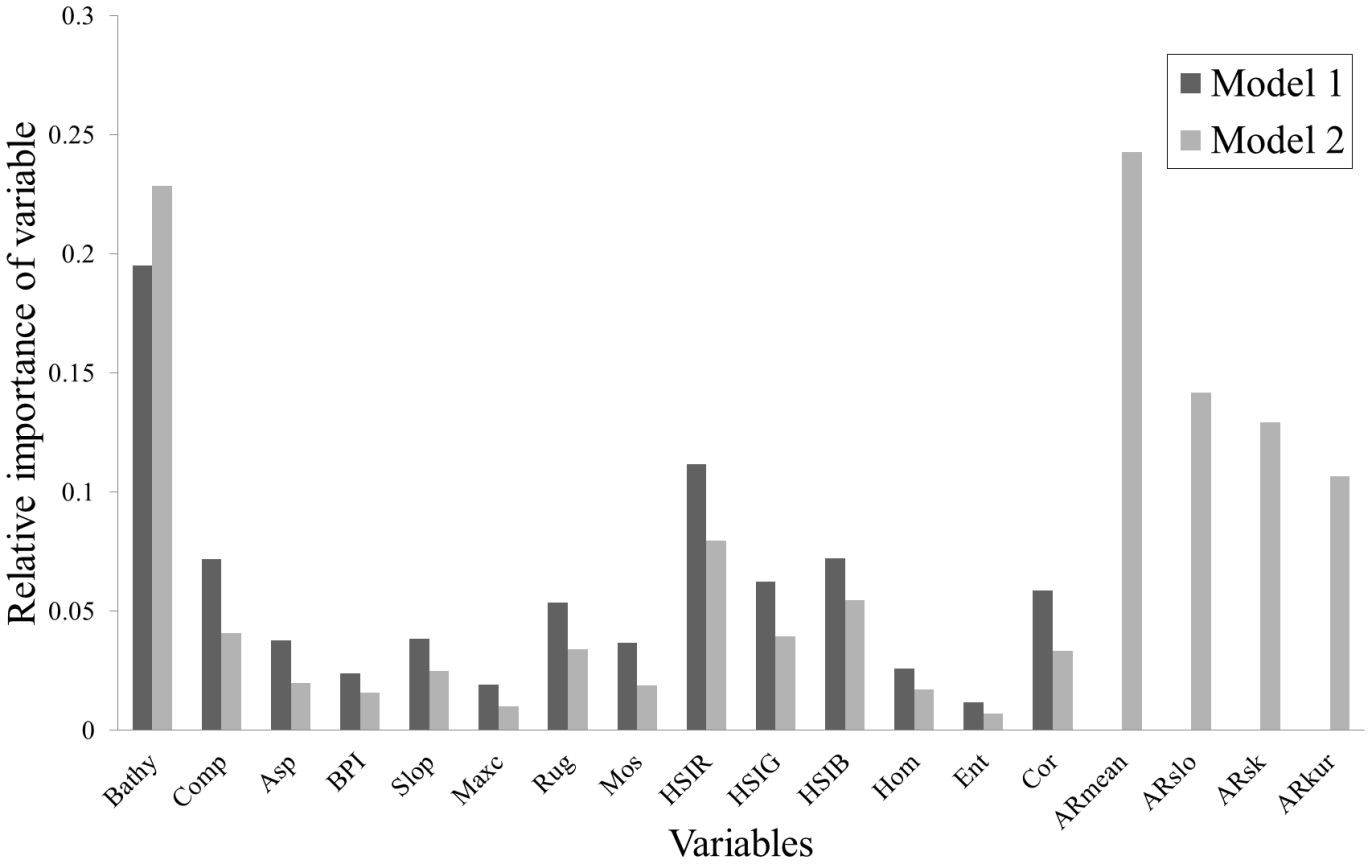
Measures of the relative importance of the variables produced from Models 1 and 2. Bathy  =  Bathymetry, Comp  =  Complexity, Asp  =  Aspect, BPI  =  Bathymetric Position Index, Slo  =  Bathymetry slope, Maxc  =  Maximum curvature, Rug  =  Rugosity, Mos  =  Backscatter mosaic, HSIR  =  Red layer of Hue Saturation and Intensity (HSI), HSIG  =  Green layer of HSI, HSIB  =  Blue layer of HSI, Hom  =  GLCM Homogeneity, Ent  =  GLCM Entropy, Cor  =  GLCM Correlation, ARmean  =  Mean of angular response, ARslo  =  Slope of angular response, ARsk  =  Skewness of angular response and ARkur  =  Kurtosis of angular response.

Bathymetry appeared the most important variable in the first model, which included all layers, except angular response derivatives (Model 1, 14 variables Figure 4). The Red HSI layer ranked second in importance. All other variables including, interestingly, the mosaic itself, were found to be of very low importance.

After the angular response derivatives were added (Model 2, 18 variables, Figure 4), bathymetry and angular response mean ranked equally as the two most important variables, closely followed by the three other angular response features (slope, skewness and kurtosis). The other variables demonstrated similar levels of low importance, as described previously for Model 1.

This order of variable importance was used to construct the subsequent four models (Models 3 to 6, see Table 3). Model 3 contained the two most important variables; bathymetry and angular response mean. The other three angular response variables (slope, skewness and kurtosis) followed with moderate importance, and were, therefore, added to generate Model 4. The three HSI layers followed with relatively little importance, and were added to generate Model 5. Finally, complexity, rugosity and GLCM correlation showed slightly more importance compared to the rest of the variables, and were added to generate Model 6.

### Model performance

**Figure 5:**
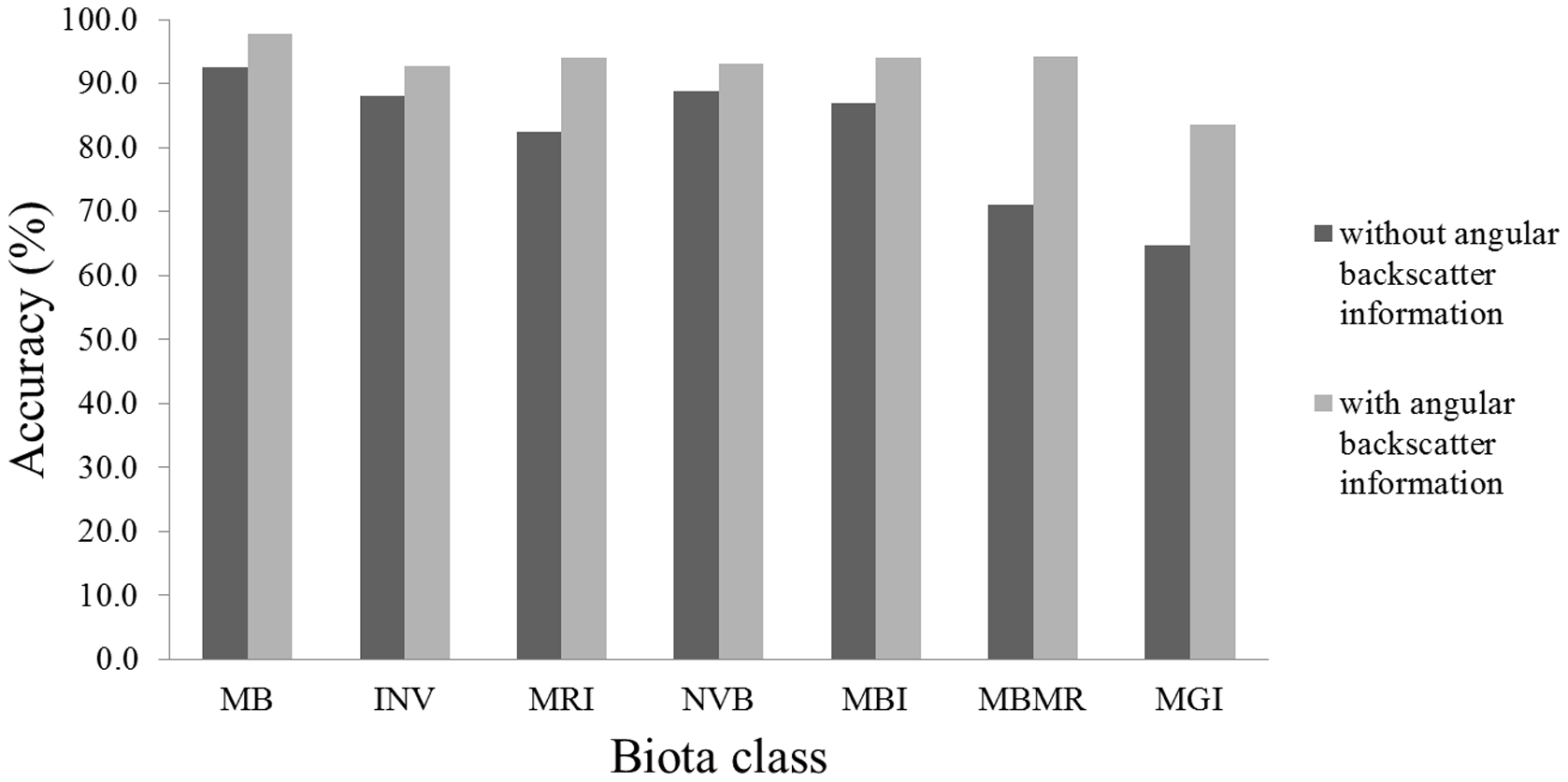
Per class accuracy (mean of user and producer's accuracy) for models 1 and 2 (with and without the backscatter angular response features). MB  =  Mixed Brown algae, INV  =  Invertebrates, MRI  =  Mixed Red algae and Invertebrates, NVB  =  No Visible Biota, MBI  =  Mixed Brown algae and Invertebrates, MBMR  =  Mixed Brown algae and Mixed Red algae, and MGI  =  Mixed Green algae and Invertebrates.

The two original models (Models 1 and 2) performed very well, obtaining overall high accuracy and kappa coefficients (Table 3). The inclusion of variables derived from the backscatter angular response from Model 1 into Model 2 increased the overall accuracy by 5.1% (88.5% to 93.6%) and the Kappa coefficient by 0.09 (0.81 to 0.90). The accuracy for all individual classes improved with inclusion of angular response derivatives, particularly for MBMR and MGI (Figure 5).

**Figure 6:**
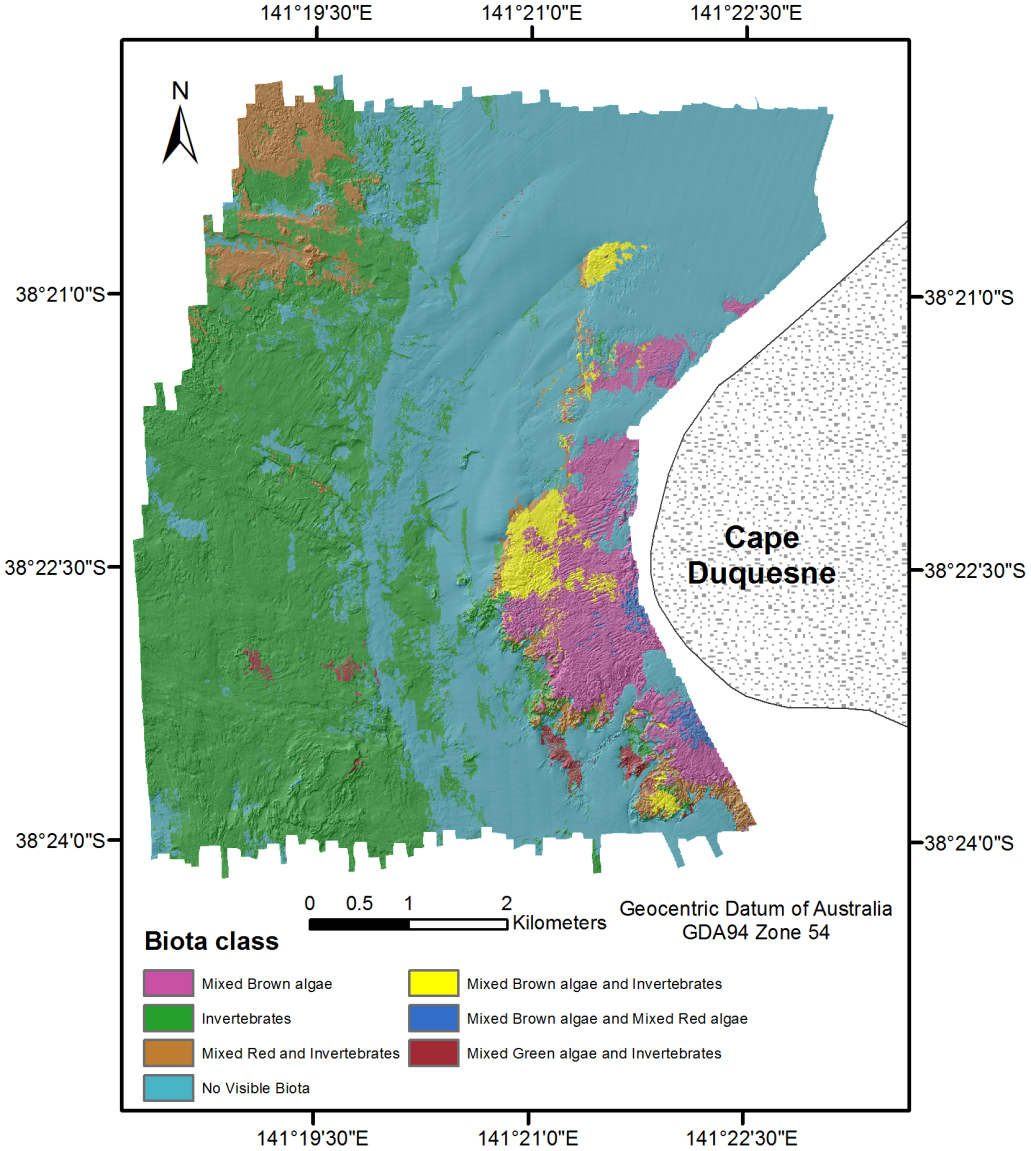
Habitat map of biota classes produced from the simplest model of variable combinations (Model 6) overlaid with the hill shaded bathymetry.

Interestingly, the simple models also achieved high accuracy, with Model 3 achieving 90.2% overall accuracy and 0.84 Kappa coefficient, despite being only driven by two variables (bathymetry and angular response mean, Table 3). The least parsimonious of the simple models (Model 6, Figure 6) performed as well as the full model (Model 2), with 93.5% overall accuracy (down by only 0.1%) and the same Kappa coefficient (0.90). Indeed, pairwise comparison of the error matrices from Models 2 and 6 indicated no significant difference between these matrices (Z = 0.29, Table 5).

**Table 5:** Pairwise comparison of error matrices between Model 2 and the four simpler models.

Pairwise combination		Z statistic	Significant/Not significant*
Model 2	Model 3	9.54	Significant
Model 2	Model 4	11.22	Significant
Model 2	Model 5	3.91	Significant
Model 2	Model 6	0.29	Not significant

Model 2 included all variables, while the simpler models contained different combinations of the variables (see Table 3). The significant level indicates whether two error matrices (i.e. from two different models) are completely different (significant), or capable of producing similar results (not significant).

*Significant at the 95% confidence interval (critical value Z = 1.96).

## Discussion

Overall, all models derived in this study achieved good accuracies, scoring between 88.5% and 93.6% (Table 3). These scores were slightly above those reached by previous studies implementing different decision tree techniques, such as CART, Quick, Unbiased, Efficient Statistical Trees (QUEST) and Classification Rule with Unbiased Interaction Selection and Estimation (CRUISE), in comparable habitats of south west Victoria, Australia [84% in 22, and 87% in 25, 80% in 26, 83% in 27]. In addition, the unique capability of RF algorithms to assess the importance of the various predictors was used to build simpler models. In the present work, the optimal model was Model 6, because it only used 11 of the 18 features derived in this research to achieve accuracy levels that were equivalent to the full model that implemented the entire set of 18 variables (Model 2). Although RF algorithms may include many variables while remaining insensitive to over-fitting [53], the use of fewer variables in the classification process has very practical benefits, in terms of gain of computer processing time and effort.

These improved results were obtained using a novel approach to integrate features derived from MBES backscatter angular response curves – which are good predictors of sediment grain-size [17] – with features describing the texture and patterns in the backscatter mosaic – which are good predictors of seafloor substrate types [54] – as well as bathymetry and its most common derived seascapes – which are good predictors of biological communities distribution [22]. To our knowledge, this constitutes the first benthic habitat mapping methodology exploiting the three main MBES data sources that are bathymetry, mosaic and angular response. In many fields linked to land mapping, improvements in classification accuracy have similarly followed from the availability of a large number of new features (i.e. spectral bands in hyperspectral remote sensing) [55], [56]. In comparison, the number of variables available for mapping in the marine realm (primarily from MBES) are severely restricted. The present study contributes to the benthic habitat mapping field by increasing the range of available acoustic variables that can be combined to characterise benthic habitats.

The main result of this study is how statistical features describing the backscatter angular response curves considerably improved class differentiation. First, the classification accuracy and the individual class accuracies were greatly improved by adding angular response features (Model 2 compared to Model 1). Secondly, the angular response mean was found to be the most important of all backscatter data derivatives, out-performing even the backscatter mosaic. In fact, all of the angular response features were ranked as more important compared to the backscatter mosaic or any of its derivatives. Finally, a simple model using only the angular response mean and bathymetry yielded higher accuracy (Model 3; 90.2%) compared to a model using mosaic, 6 mosaic derivatives, bathymetry and 6 bathymetry derivatives (Model 1; 88.5%). These results suggest that the methodology presented here deriving statistical features from the backscatter angular response, successfully captured the characteristics of backscatter variation at moderate incidence angles, which are known to successfully discriminate between seabed types [18] or benthic communities [41]. The fact that the angular response mean has the appearance of a de-noised version of the backscatter mosaic (Figures 1 and 3) probably also contributed to the success of this feature. In effect, the speckle and nadir noise commonly displayed in backscatter mosaics are likely to be responsible for errors in the classification process, and, hence, hinder the predictive power of the backscatter mosaic. By effectively enhancing the meaningful backscatter contrasts between the small regions of the study site, while removing the noise and maintaining the mosaic spatial resolution, the methodology used to derive the angular response mean in this study may be viewed as creating an improved version of the backscatter mosaic.

The other three angular response features (slope, skewness and kurtosis) were found to be more important compared to the features derived from bathymetry and the mosaic, but were less relevant compared to the angular response mean. Interestingly, the addition of these three features to the model using only bathymetry and angular response mean decreased the classification accuracy (from Model 3; 90.2%, to Model 4; 89.7%). The inconsistency between the relevance of these features and the decrease in accuracy following their addition warrants future investigation. At present, it may be assumed that the unique areas that angular response skewness and angular response kurtosis appeared to single out in the south-western part of the study site (Figure 3) may be irrelevant in terms of habitat differences, and might have caused some inconsistencies in the final habitat map. The angular response slope probably does not contribute to this problem as it is more similar to the angular response mean and the original mosaic. Incorporating only the slope with bathymetry and the angular response mean might have produced a more successful model compared to Model 4.

Regardless of the success of using angular response features in this study, bathymetry was found to be the single-most important habitat predictor across all models. The high accuracy of the simplest model using only bathymetry and angular response mean in this study (Model 3), and the low correlation between these two features, indicates that they contain very different and complementary information to predict benthic habitats. These results reinforce the argument that benthic habitat mapping efforts should not be based on backscatter data information alone [57].

Finally, a number of observations may be made for the less relevant features in this study. Although bathymetry and the angular response mean were undeniably the most important features (Model 3), they did not produce the best accuracy alone. The highest accuracy was achieved with a mix of bathymetry and mosaic derivatives (HSI layers, rugosity, complexity and GLCM correlation; Model 6), indicating that these minor features described subtle variations in terrain complexity across biotic habitat types. The topographic features other than rugosity and complexity (i.e. slope, aspect, maximum curvature and bathymetric position index) did not make a significant contribution to this study, which contrasted with the findings of previous works on the distribution of habitats of shallow water mobile species [58], [59].

The approach presented in this study and its accuracy results are dependent on two main factors: the types of biological benthic habitat present in the study site and the scheme originally used to classify the ground-truth data. Soft sediments are the dominant seabed types in the present study site; the shallower north eastern areas inside Discovery Bay were largely composed of fine, well sorted sand flats with some fine rippling, while sediments composing the deeper areas to the west of the site tended to be coarser and formed into broad (>40 cm), well defined sand waves. These sediment dominated areas seldom had visible epifauna present and were therefore clustered together under the “no visible biota (NVB)” biotic habitat class, according to the classification scheme chosen for the study. Yet, previous studies investigating infaunal communities of eastern Victoria suggested that these regions contain highly diverse temperate infaunal assemblages [60], [61]. Although assigned to only one class in this study, researchers have found that unconsolidated, sandy sediments can be further allocated to sub-classes which are acoustically distinct based on sediment size, surface morphology, and compactness [7], [62]–[65]. Furthermore, there is evidence to suggest that distinct assemblages of fish and invertebrates exist within and between biotopes defined by grain size and ripple characteristics [66], [67]. The present study did not identify such potential diversity due to its classification scheme focusing on epifauna biota assemblages. This limitation emphasizes how model accuracy results need to be appreciated in the context of the ground-truth data being used and the classification scheme that was applied to them. However, the approach presented in this study is very flexible and could be similarly applied to soft-sediment classification schemes to advance the benthic characterisation of sediment types in benthic habitat mapping studies.

## Conclusions

The overall high accuracy of all models in this study indicated that the suggestion to integrate angular response features with bathymetry and backscatter mosaic and their derivatives is sound and effective. High model accuracy was obtained using just bathymetry and angular response mean, with this further increasing following addition of other angular response features and features considered relevant based on RF algorithm analyses. While the angular response mean over mid-range incidence angles proved an important contributor to improving our prediction of benthic classes, the other angular response features (slope, skewness and kurtosis) produced more mixed results. It is anticipated that this methodology will be applied to other datasets to assess whether these three features should be conserved or abandoned, and if other angular features should be developed.

The results also confirmed that bathymetry remains the most important predictor of marine biotic habitats, and highlighted that some bathymetry and mosaic derivatives were rather irrelevant to the classification process. These conclusions were reached by using a classification algorithm that allowed the model variables to be ranked by the importance of their contribution to the resulting model. Since the importance of any given feature might depend on the habitats present in the study area, we strongly recommend the use of RF algorithms, or other classifiers that have this additional capability.

## Supporting Information

Supplementary S1 **A zip file containing all Matlab codes used in this paper to construct mean angular response curves from homogeneous regions using the “proc” files (processed files from the CMST MB Process software) and user-set polygons (ArcMap shapefile format).** Users need to unzip this file and add all codes to their local Matlab path. The main function is ‘inpoly_plosone_v1.m’ (to see full instructions, type ‘help inpoly_plosone_v1’ in the Matlab Command Window). Running this code in Matlab will display a Matlab graphical user interface to run the analysis (successfully tested on Matlab R2013a 32-bit).
(ZIP)
